# Guidelines of inclusive architecture design for autism spectrum disorder: What is new?

**DOI:** 10.1192/j.eurpsy.2024.272

**Published:** 2024-08-27

**Authors:** E. Abdelmoula, N. Bouayed Abdelmoula, B. Abdelmoula

**Affiliations:** ^1^LR AMC, Ecole Doctorale Sciences et Ingénierie Architecturales (ED-SIA), Tunis; ^2^Genomics of Signalopathies at the service of Precision Medicine - LR23ES07, Medical University of Sfax, Sfax, Tunisia

## Abstract

**Introduction:**

Autism spectrum disorder (ASD) is a complex neuro-developmental condition. According to the Diagnostic and Statistical Manual of Mental Disorders (DSM-5), restricted interests and repetitive behaviors and difficulties with social communication and interaction characterize ASD. Different ways of learning, moving, or paying attention are related to the degree of impairments. By reducing environmental and social obstacles in school, work, and other areas of life, architecture could play a pivotal role in helping people on the spectrum become more independent and acquire more abilities.

**Objectives:**

The aim of this study was to outline the recommendations and guidelines of the inclusive architecture design for ASD.

**Methods:**

We conducted a comprehensive review of the scientific literature using the following keywords: inclusive design, architecture, autism or ADS.

**Results:**

Our research found that the Autism ASPECTSS design index reported in 2013 by Magda Mostafa from Canada, which was based on the sensory design theory, is the world’s first set of evidence-based design guidelines for managing built environments to serve ADS individuals interaction, particularly in schools and workspaces. ASPECTSS conceptual framework delineate seven design concepts: acoustics, spatial sequencing, escape space, compartmentalization, transition spaces, sensory zoning, and safety. In 2023, the same author published an autism friendly design guide for the world’s first autism-friendly university. This guide is characterized by a better understanding of human-centered design and advocates beyond the mere inclusion, aspiring to a state where the boundaries between ‘normal’ and ‘special’ are blurred in order to treat all users as human beings with equal rights, thus calling for equal opportunities beyond the ADS spectrum.

**Conclusions:**

With such well-established conceptual framework, it is nowadays imperative to expand our buildings in cities, schools, workplaces, hospitals, and public areas using the guidelines of autism-friendly environments. These buildings will enhance our individual and social well-being.

**Disclosure of Interest:**

None Declared

